# Editorial: Therapeutic strategies for drug-induced liver injury: Review of the current literature

**DOI:** 10.3389/fphar.2022.1094732

**Published:** 2022-11-24

**Authors:** Mercedes Robles-Díaz, Judith Sanabria-Cabrera, Einar S Björnsson

**Affiliations:** ^1^ Unidad de Gestión Clínica de Aparato Digestivo, Instituto de Investigación Biomédica de Málaga-IBIMA, Hospital Universitario Virgen de la Victoria, Facultad de Medicina, Universidad de Málaga, Centro de Investigación Biomédica en Red de Enfermedades Hepáticas y Digestivas (CIBERehd), Málaga, Spain; ^2^ Servicio de Farmacología Clínica, Instituto de Investigación Biomédica de Málaga-IBIMA, Hospital Universitario Virgen de la Victoria, Universidad de Málaga, Málaga, Spain; ^3^ Faculty of Medicine, University of Iceland, Reykjavik, Iceland; ^4^ Department of Gastroenterology, Landspitali University Hospital Reykjavik, Reykjavik, Iceland

**Keywords:** management in DILI, DILI treatment, DILI prevention, novel therapies in DILI, corticosteroids in DILI, N-acetylcysteine in DILI, ursodeoxycholic acid in DILI

## Introduction

*Idiosyncratic* drug‐induced liver injury (DILI), although an infrequent entity, was the cause of acute liver failure in 13%–15% of cases in reports from the United States and Sweden (Ostapowicz et al., 2002; Wei et al., 2007). Its management is based on the withdrawal of the responsible drug with liver transplantation for more severe/fulminant cases. Many drugs have been used in DILI treatment without clear evidence of efficacy. This Research Topic aims to carry out an in-depth review of the literature to investigate the role of agents most commonly used in the treatment and prevention of DILI as well as analyze the current knowledge in order to design an appropriate controlled randomized clinical trial for DILI management. It includes seven articles in which studies addressing the role of ursodeoxycholic acid (UDCA), corticosteroids, N-Acetylcysteine (NAC), immunotherapy and gene therapy as well as, other novel therapies in the treatment and or prevention of DILI.

## Drugs for treating and preventing drug‐induced liver injury

In the systematic review of Robles-Diaz et al. about of the role of UDCA in DILI, 24 case reports of DILI (with 30 patients), three prospective studies (one being a clinical trial) and one retrospective study evaluating UDCA in DILI treatment were identified. Additionally, a case report, a pilot study, two randomized clinical trials (RCT) and one retrospective study were found evaluating UDCA in DILI prevention. Twenty-four DILI cases and 6 clinical studies reported benefits of UDCA therapy in time required for total bilirubin (TBL) decrease, normalization or reduction of liver test parameters, or avoiding increase in aminotransferases when used in prevention. Unfortunately, we cannot reach a firm conclusion about the role of UDCA in DILI management and prevention due to the limitations in the design of the published studies.

Bjornsson et al. analyzed the literature with regards to the potential role of corticosteroids in DILI management, with most studies analyzing the effects in moderate/severe DILI and suggesting beneficial effects, except for in drug-induced fulminant ALF. The best response was found in patients with drug-induced autoimmune-like hepatitis (DI-AILH). Patients with liver injury due to checkpoint inhibitors (CPIs) also seem to respond to corticosteroids. However, some of these patients recovered spontaneously and some were steroid unresponsive. Similar to what occurs for UDCA, the lack of well-designed controlled RCT does not allow firm conclusions to be drawn on the efficacy of corticosteroids in DILI.

N-Acetylcysteine in non-acetaminophen (non-APAP) DILI prevention and treatment was reviewed by Sanabria-Cabrera et al. This systematic review included eleven studies, of which eight were RCT. Two out of three RCT and one observational study concluded effectiveness of NAC in prevention of anti-tuberculosis drug-related DILI. NAC for treatment of non-acetaminophen DILI was part of studies evaluating NAC in ALF of different aetiologies. Among the benefits of NAC were improvement of liver transplant (LT)-free survival, improved liver parameters and reduced length of hospital stays, however, the limitations detected across studies once again prevent clear conclusions.

With regards to NAC in APAP DILI however, Licata et al. found improved prognosis and reduced mortality when administered within 8–24 h from APAP overdose in 34 studies with almost 20,000 patients. Although NAC dose regimen and treatment length varied among the studies, all showed benefits.

A general review of agents used for treatment or prevention of DILI was carried out by Li et al. They classified hepatoprotective drugs (NAC, Glutathione, Bicyclol, Polyene phosphatidylcholine, Silymarin, Glycyrrhizin acid preparation), drugs for cholestasis (UDCA, Cholestyramine and S-adenosylmethionine), immunosuppressants (Glucocorticoids) and specific treatment agents (L-carnitine, Anticoagulants).

Randomized clinical trials were only found for evaluations of hepatoprotective drugs. Treatment with magnesium isoglycyrrhizinate (MgIG) in DILI seems to increase normalization rate of ALT and AST when comparing with tiopronin treatment. Bicyclol showed higher reduction of ALT than polyene phosphatidylcholine and was superior to silybin and diammonium glycyrrhizinate in DILI treatment. Sylimarin was observed to improve ALT alterations better than UDCA in DILI in one RCT, however, these results were not confirmed in other RCTs compared with placebo. Therefore, the definite therapeutic effects of these drugs are still to be confirmed by prospective randomized and controlled studies.

In the systematic review of novel therapies in DILI, *by* Benic et al., twenty-eight articles were included, and among them eight RCT. They found the following agents: bicyclol, *Paeoniae radix rubra*, calmangafodipir, livina-polyherbal preparation, cytisin amido phospate, picroliv, fomepizole, magnesium isoglycyrrhizinate, plasma exchange, and S-adenosylmethionine. Among them, only bicyclol and MgIG showed low to moderate evidence of liver profile improvement compared to phosphatidylcholine and placebo, respectively.

Delire et al. reviewed the management of DILI due to Immunotherapy (CPIs). Temporary CPI withdrawal has been proposed in case of grade 2 and 3 hepatitis and permanent discontinuation for grade 4 hepatitis. Corticosteroids can be administered to patients with grade 2 or more severe liver injury. For those corticosteroid refractory cases, second-line immunosuppressive drugs have been proposed (mycophenolate mofetil).

There is only limited information about safety in CPIs rechallenge after temporary withdrawal, although it seems an appropriate option in many cases. As a matter of fact, the guidelines for management of liver toxicity due to CPIs are mainly based on expert opinions and case series.

In the context of gene therapy, in clinical trials for haematological diseases incidences of liver toxicity may occur in up to 60% Delire et al. (9). Corticosteroids and other immunosuppressants have been used for treatment as well as prophylaxis. The benefit of corticosteroid treatment seems clear, but is also associated with reduced gene therapy activity. Data in this field is very limited and the majority remains investigational.

## Concluding remarks

Treatment in DILI remains a challenge. DILI with different phenotypes and different culprit drugs could benefit from a different treatment strategy. Except for NAC in APA-induced liver injury with demonstrated efficacy in the improvement of liver damage, only moderate or very limited evidence of benefits has been shown for other treatment options that have been tried (bicyclol, MgIG, corticosteroids). Further well-designed controlled RCT are needed in order to find therapies that might alter the natural history of DILI.
